# A speech compression method without utilizing signal prediction

**DOI:** 10.1177/20416695251340236

**Published:** 2025-05-21

**Authors:** Ikuo Matsuo, Kazuo Ueda, Yoshitaka Nakajima

**Affiliations:** 1 13284 Tohoku Gakuin University, Sendai, Japan; 2 12923 Kyushu University, Fukuoka, Japan

**Keywords:** speech signal, low-bit-rate speech coding, vocoding, intelligibility, periodicity, peak-clipping, amplitude envelope

## Abstract

Previous speech compression methods for practical purposes had been based on signal prediction, taking the auditory functions into account but overlooking features specific to speech signals. A new method was developed in which amplitude envelopes in four frequency bands corresponding to spectral factors common to different languages were used to modulate infinitely peak-clipped signals, which also had been revealed to contain useful linguistic information. In a pilot experiment, intelligibility reached ~80% with limited information of only 2,400 bits per second (bps), whereas the bit rate of the original signal was 256,000 bps. This algorithm preserves the naturalness of speech and is easy to grasp intuitively.

## How to cite this article

Matsuo, I., Ueda, K., & Nakajima, Y. (2025). A speech compression method without utilizing signal prediction. *i–Perception, 16*(0), 1–5. https://doi.org/10.1177/20416695251340236

Signal predictions, such as linear predictive coding (LPC; e.g., Kohler, 1997; Schroeder & Atal, 1985), have been widely used to encode speech waveforms with reduced bit rates. However, even the state-of-the-art code-excited linear prediction (CELP) requires more than 4,000 bits per second (bps), and thus developing a speech encoding technique that requires less than 4,000 bps had been considered challenging (McCree, 2008). We propose an algorithm called *1-bit vocoding* that conveys speech with bit rates lower than 4,000 bps, while preserving intelligibility and naturalness.

**Figure 1.**
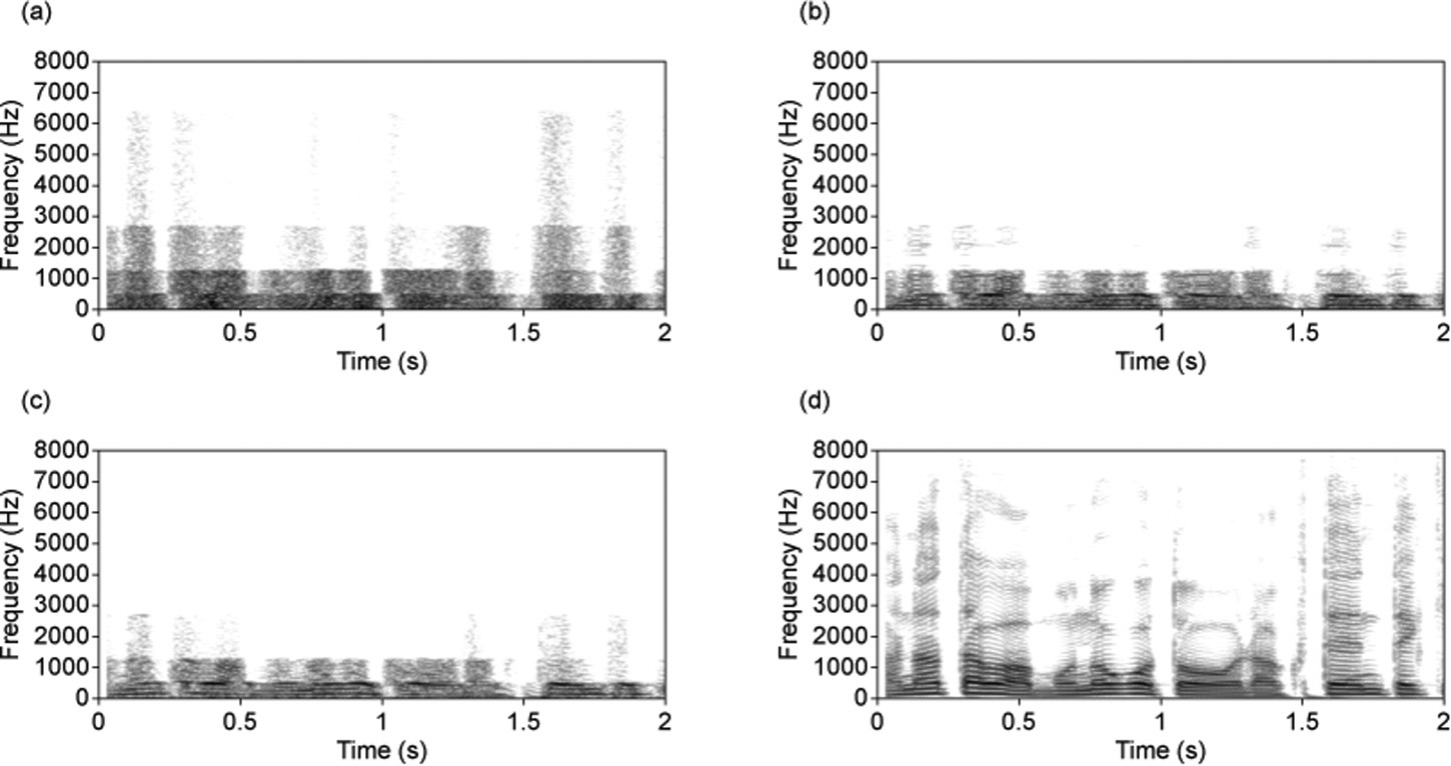
Narrowband spectrograms showing portions of the Japanese speech stimulus samples and audio demonstration materials (in Japanese in online Movie 1 and in English in online Movie 2). (a) A noise-vocoded speech stimulus (Sound 1, 800 bps), (b) a proposed 1-bit vocoded speech stimulus (Sound 2, 2,400 bps), (c) a proposed 1-bit vocoded speech stimulus (Sound 3, 4,800 bps), (d) the original speech sample (Sound 4, 256,000 bps). The amplitude envelopes of the noise-vocoded and the 1-bit vocoded speech stimuli were sampled at a 25 Hz sampling rate and quantized at 8 bits.

Amplitude envelopes in the four frequency bands common to several different languages (Ueda & Nakajima, 2017) provide enough information to make speech signals reasonably intelligible (Ellermeier et al., 2015). Figure 1(a) illustrates amplitude-modulated noises in these four frequency bands. Sound 1 (800 bps) in online Movie 1 (Japanese) and online Movie 2 (English) demonstrates that combined amplitude-modulated noises are intelligible (Shannon et al., 1995; Ueda et al., 2018). However, this procedure, noise-vocoding, makes the speech quality very unnatural. Aiming at recovering naturalness, we adopt infinitely peak-clipped speech instead of noise, as it gives some information on vowels and consonants (Licklider & Pollack, 1948; Sakai & Inoue, 1960) [Figure 1(b)]. Naturalness recovers as in Sound 2 (online Movies 1 and 2) (2,400 bps). This happened without training listeners.

**Figure 2.**
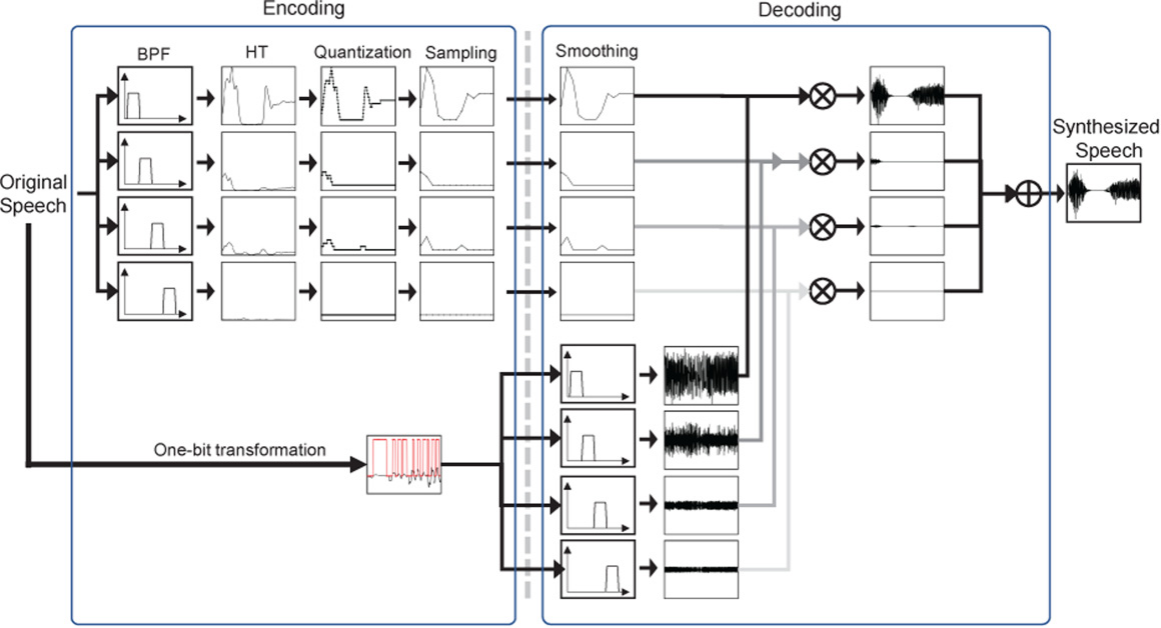
A block diagram showing the encoding and decoding processes using the proposed *1-Bit Vocoder*. The encoding process consists of two sets of devices, extracting amplitude envelopes with the upper set and coarse periodicity information with the lower set. A speech signal is divided into four frequency bands with BPFs in the upper set of the diagram. The amplitude envelopes are extracted with the HT, quantized at 6, 8, or 16 bits, and sampled at 25 or 40 Hz rates. The 1-bit transforming process is run in the lower set by converting the positive samples in the original waveform into “1” and all the other samples into “0”. smoothed amplitude envelopes are multiplied by the filtered 1-bit transformed signals at the decoding stage.

*Note*. BPF = band-pass filter; HT = Hilbert transformation.

As in Figure 2, the encoding process consists of two sets of devices, extracting band-pass filtered amplitude envelopes with the upper set and coarse periodicity information with the lower set. A speech signal is divided into four frequency bands in the upper set, and an amplitude envelope is extracted in each frequency band with the Hilbert transformation (HT). The extracted amplitude envelopes are quantized at 6, 8, or 16 bits, and sampled at 25 or 40 Hz rates. The bit rate for the four amplitude envelopes can be as low as 600 bps in total (6 bits at a 25 Hz rate in four frequency bands). In the lower set, the coarse periodicity is conveyed with a 1-bit transformation, that is, converting positive samples in the waveform into 1 and all other samples into 0 at 800, 1600, 2000, 4000, and 16,000 per second, with the minimum additional cost for processing and amount of information compared with the original noise-vocoding. The bit rate for this coarse periodicity signal is the same as the sampling rate. The speech bit rate for the 1-bit vocoding technique is the sum of the bit rates for the amplitude envelopes and the coarse periodicity. Smoothed amplitude envelopes are multiplied by the filtered 1-bit transformed signals at the decoding stage.

**Figure 3.**
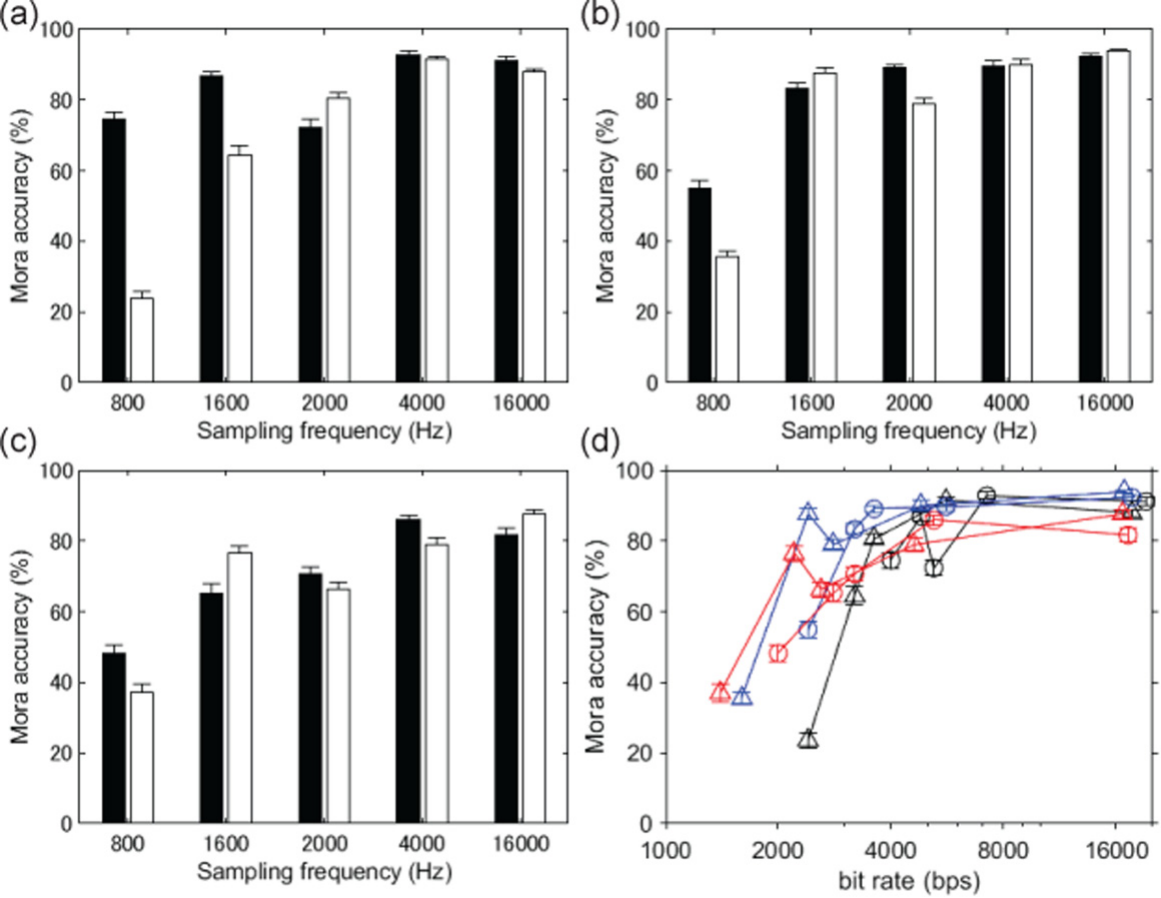
Results of the pilot experiment showing mora (a unit in Japanese shorter than or equal to a syllable) accuracy with 12 Japanese native participants. (a–c) Mean percentages of mora accuracy as functions of sampling frequency. Bit depth: (a) 16 bits, (b) 8 bits, and (c) 6 bits. The sampling rates for amplitude envelopes were 25 (white bars) and 40 Hz (black bars). (d) Mora accuracy is indicated as a function of the bit rate of encoding. The circles and triangles represent 25 and 40 Hz rates for amplitude envelope sampling. The black, blue, and red curves show 16, 8, and 6 bits for quantization. Error bars reflect standard errors of the mean (SEMs).

For a pilot experiment, 30 everyday sentences in Japanese spoken by a male speaker were selected from the NTT-AT Multilingual Speech Database 2002 (NTT-AT, Kawasaki, Japan; recorded with a 16 kHz sampling rate and 16 bit linear quantization). All stimuli were produced with the 1-bit vocoding technique. Each sentence was presented only once to each listener. These results with 12 Japanese native listeners showed that intelligibility of about 80% could be attained even for speech stimuli compressed to 2,400 bps [Figure 3 (b), at the 1600 Hz sampling frequency]. For Sound 2 [as in Figure 1(b), in Japanese in online Movie 1 and in English in online Movie 2], amplitude envelopes were sampled at a 25 Hz rate and quantized at 8 bits for four frequency bands [20–510, 510–1270, 1270–2700, and 2700–6400 Hz, based on Ueda and Nakajima (2017)], and coarse periodicity was obtained at a sampling rate of 1600 Hz.

Speech signals severely degraded by removing periodicity and reducing frequency resolution are hard to hear (Sound 1 in online Movies 1 and 2). Acoustic signals resynthesized without periodicity sound very unnatural, especially for the first listening. Adding coarse (low bit) periodicity cues recovers the naturalness (Sound 2 in online Movies 1 and 2) and improves the speech quality. This demonstration clarifies that the temporal periodicity, as observed in infinitely peak-clipped speech (Licklider & Pollack, 1948), plays a role different from that of the amplitude envelopes in the four frequency bands corresponding to spectral factors of speech (Ueda & Nakajima, 2017), leading to an intuitively simple speech-compressing algorithm requiring bit rates well below 4,000 bps, while enabling reasonable communication.

## Supplemental Material

**Figure:** Video 1.

**Figure:** Video 2.
